# Vietnamese Version of the General Medication Adherence Scale (GMAS): Translation, Adaptation, and Validation

**DOI:** 10.3390/healthcare9111471

**Published:** 2021-10-29

**Authors:** Thao Huong Nguyen, Hoa Van Truong, Mai Tuyet Vi, Katja Taxis, Thang Nguyen, Kien Trung Nguyen

**Affiliations:** 1 Department of Clinical Pharmacy, Faculty of Pharmacy, University of Medicine and Pharmacy at Ho Chi Minh City, Ho Chi Minh City 700000, Vietnam; huongthao0508@gmail.com (T.H.N.); truongvanhoa885@gmail.com (H.V.T.); 2 Department of Pharmacology and Clinical Pharmacy, Can Tho University of Medicine and Pharmacy, Can Tho City 900000, Vietnam; maivivi127@gmail.com; 3 Groningen Research Institute of Pharmacy, University of Groningen, 9713 AV Groningen, The Netherlands; k.taxis@rug.nl; 4 Department of Physiology, Can Tho University of Medicine and Pharmacy, Can Tho City 900000, Vietnam

**Keywords:** translation, adaptation, validation, medication adherence, diabetes, Vietnamese

## Abstract

Background: We aimed to translate, cross-culturally adapt, and validate the General Medication Adherence Scale (GMAS) into Vietnamese. Methods: We followed the guidelines of Beaton et al. during the translation and adaptation process. In Stage I, two translators translated the GMAS to Vietnamese. Stage II involved synthesizing the two translations. Stage III featured a back translation. Stage IV included an expert committee review and the creation of the pre-final version of the GMAS, and in stage V, pilot testing was conducted on 42 Vietnamese patients with type 2 diabetes. The psychometric validation process evaluated the reliability and validity of the questionnaire. The internal consistency and test–retest reliability were assessed by Cronbach’s alpha and Spearman’s correlation coefficients. The construct validity was determined by an association examination between the levels of adherence and patient characteristics. The content validity was based on the opinion and assessment score by the expert committee. The Vietnamese version of the GMAS was created, including 11 items divided into three domains. There was a good equivalence between the English and the Vietnamese versions of the GMAS in all four criteria. Results: One hundred and seventy-seven patients were participating in the psychometric validation process. Cronbach’s alpha was acceptable for all questionnaire items (0.817). Spearman’s correlation coefficient of the test–retest reliability was acceptable for the GMAS (0.879). There are significant correlations between medication adherence levels and occupation, income, and the Beliefs about Medicines Questionnaire (BMQ) score regarding construct validity. Conclusions: The Vietnamese version of GMAS can be considered a reliable and valid tool for assessing medication adherence in Vietnamese patients.

## 1. Introduction

Medication non-adherence can cause treatment failure. In the USA, medication non-adherence was responsible for 30–50% of treatment failure, increasing hospitalization (10%) and mortality rates (125,000 cases per year) [1]. This indicates that medication adherence positively affects treatment targets and reduces the risk of adverse events. It is known that diabetes is a chronic disease and upward trend. According to the statistics of the International Diabetes Federation in 2019, there were 463 million people with diabetes prevalence, increasing to 578 million by 2030 and 700 million by 2045 [2]. Diabetes is a severe disease that requires long-term treatment to achieve glycemic, HbA1c control, and minimize complications [3]. Reasonable glycemic and HbA1c (Hemoglobin A1C) control reduces the mortality rate and the risk of microvascular and macrovascular complications [4].

Nevertheless, recent data indicate that diabetes has not been well controlled. A study of 15,404 patients with type 2 diabetes in China (2016) or other studies in Sudan (2019) and India (2019) show that about 80% of patients did not achieve glycemic targets [5,6,7]. In these studies, one of the reasons for the lack of glycemic control is medication non-adherence. Medication adherence is an integral factor in helping diabetic patients achieve treatment goals besides lifestyle modification and pharmacologic therapy.

Assessing medication adherence in patients with chronic diseases (hypertension, diabetes, dyslipidemia, etc.) plays a significant part in taking interventions to advance adherence and therapeutic effect for patients. There are many methods of measuring medication adherence, and each method has different advantages/disadvantages [8]. However, indirect assessment with scale is widely used in countries including Vietnam, such as the Eight-Item Morisky Medication Adherence Scale (MMAS-8) [9]. Undeniably, MMAS-8 has drawbacks such as unevaluated non-adherence due to financial constraints, difficulty contacting the author during the study process, or copyright issues when researchers publish results. Therefore, developing a new scale is necessary to overcome the above limitations for application in research conditions in Vietnam.

The GMAS was developed by Atta Abbas Naqvi et al. (2018) to measure patients’ medication adherence with chronic diseases. Currently, the GMAS, which surmounts the demerits of MMAS-8, does not have a Vietnamese version. The research team was permitted to use the GMAS by the author and his collaboration team. We conducted the study to translate, adapt, and validate the GMAS in patients with type 2 diabetes, which is one of the leading causes of death in Vietnam.

## 2. Materials and Methods

### 2.1. Study Population

The study was conducted at a hospital in Ho Chi Minh City from January 2020 to August 2020. The study selected type 2 diabetic patients who met the following criteria: 18 years or older, on outpatient treatment with at least one diabetes drug six months ago. We excluded patients with one of the following criteria: (patients) are pregnant or intend to become pregnant, have an acute illness, lack access to Vietnamese, decline participation in the study or do not complete at least one question of the scale used in the survey form, and participated in a study related to the medication adherence within the last six months. In this study, regarding test–retest reliability, we excluded patients who refused to participate or could not be contacted at the second phone interview 14 days away from the first interview because the purpose of the second interview was to assess the stability of the scale. In addition, we assessed patient compliance based on the scale’s total score, and the above patients had yet to be evaluated, so we could not accurately determine their level of compliance and excluded them from our study.

### 2.2. Study Design

We conducted translation and cross-cultural adaptation of the GMAS following the guidance of Beaton et al. [10,11].

#### 2.2.1. Stage I—Initial Translation

The GMAS was translated from English to Vietnamese by two translators who spoke Vietnamese as their mother tongue and were capable of good English comprehension. The first translator had medical expertise and comprehended the assessment purposes and scale concepts. The second translator did not possess medical knowledge and did not comprehend the assessment purposes and scale concepts. The result of the procedure created two translations called T1 and T2.

#### 2.2.2. Stage II—Synthesis of Two Translations

The third translator, having experience in methodology, synthesized two translations (T1 and T2). This stage was finished with the T12 version of the GMAS.

#### 2.2.3. Stage III—Back Translation

The T12 version of the GMAS was translated from Vietnamese to English by two translators who did not know the original version, did not have medical expertise, and were fluent in Vietnamese and English. Finally, this process created two back translations called BT1 and BT2.

#### 2.2.4. Stage IV—Expert Committee Review

The expert committee included ten members: five translators (in stages I, II, and III), a methodologist, two endocrinologists with experience in research, a clinical pharmacist, and the author of the GMAS.

Stage 4 was conducted through the following steps:

*Step 4.1.*  *Assessing the GMAS:* The expert committee evaluated the equivalence of each question in the translations compared to the original independently. The researcher designed the assessment form, including all versions of the GMAS and the content that needed adjustment. The evaluation criteria included semantic equivalence, idiomatic equivalence, experiential equivalence, and conceptual equivalence—rating scale: 1 point if there is equivalent and 0 points if there is no equivalent.
*Step 4.2.*  *Adjustment of questions that did not gain equivalent score:* The questions did not achieve absolute equivalence (10/10) for four criteria, and suggestions for adjusting the content of the scale (proposed by members of the committee in Step 4.1) were synthesized by the researcher. They were sent to the members of the committee, who reviewed and adjusted them.
*Step 4.3.*  *Assessing the GMAS after adjustment:* The questions after adjusting were evaluated for the second equivalence by the expert committee as the four criteria and scale in Step 4.1. After this step, the pre-final version of the GMAS was generated and used for pilot testing.

#### 2.2.5. Stage V—The Pilot Testing

A minimum of 30 patients was required to participate in this stage. The purpose of the pilot testing was to discover the clarity and comprehensibility of each question. Patients evaluated the expression of each question in the pre-final version on a scale from 0 (unclear) to 10 (very clear). The researcher recorded explanations and suggestions of patients for each question. After that, the committee reviewed and adjusted the questions with an average score of ≤9 to create a complete Vietnamese version of the GMAS.

### 2.3. Materials

The GMAS was developed by Atta Abbas Naqvi et al., and it was initially written in Urdu [12]. However, the GMAS was translated and validated to the English version by the author and their collaborators [13]. In this study, we used the English version of the GMAS because it is easier to access. The author has permitted the research team to use and collaborate in the translation and validation of the GMAS for the Vietnamese version. The scale comprises 11 questions divided into three domains: non-adherence due to patient behavior, non-adherence due to other disease and pill burden, and non-adherence due to financial constraints. Interviewees answer each question with four options based on the Likert scale (including always, mostly, sometimes, and never, which correspond to 3, 2, 1, and 0 points). Cumulative medication adherence was assessed in two ways:

- Option one: medication adherence is divided into two levels, including adherence (≥27 points) and non-adherence (<27 points).
- Option two: medication adherence is divided into five levels, including high adherence (30–33 points), good adherence (27–29 points), partial adherence (17–26 points), low adherence (11–16 points), and poor adherence (0–10 points).

In addition, the GMAS describes the level of medication adherence in each domain.

### 2.4. Psychometric Validation of the GMAS

#### 2.4.1. Sample Size

In the translation and validation of the English version of the GMAS, the researchers selected the sample size based on the number of patients needed to respond to each question. In this study, we chose sample size as the author’s way and related guidelines. The GMAS had 11 questions. Thus, with a response rate of 1:10 (each question required ten patients), the sample size was 110 patients [12,13,14,15]. For increased accuracy, we chose 165 patients (response rate 1:15). In Terwee, Comrey, and Lee’s guidelines, the appropriate sample size was a minimum of 50 or 100 patients [16,17]. Therefore, 165 patients were suitable.

#### 2.4.2. Psychometric Validation

The GMAS was assessed for its reliability and validity. The internal consistency was shown by Cronbach’s alpha coefficients. For test–retest reliability, the same patients with type 2 diabetes answered the questions two times. The test–retest reliability was assessed over Spearman’s correlation coefficients based on the repetition between the first and second interviews (14 days apart). Content validity was based on the opinion and assessment score of the equivalence between the translations and the original of the GMAS from the expert committee. Construct validity was assessed by determining whether the relationship between the questions, sections, and scale content was consistent with the study’s hypotheses about the score outcomes of different population groups. A scale would have construct validity if it made a significant difference between medication adherence and patient factors (age, sex, occupation, etc.) [18,19]. The study of Quynh Nguyen Phuong Huynh et al. also evaluated the validity of the construct according to the above method [20].

### 2.5. Statistical Analysis

Data were statistically analyzed using SPSS version 25.0 (IBM, Armonk, NY, USA). Data were presented by categorical variables and continuous variables. Descriptive statistics were used to describe the demographic and disease characteristics of the patients. The corrected item-total correlation, Cronbach’s alpha (α), and Spearman’s correlation coefficients were used to evaluate reliability. The construct validity was based on Chi-square, Mann–Whitney, and Kruskal–Wallis tests.

### 2.6. Ethical Approval

Patients’ information is for research purposes only, not for any other purpose. The study was approved by the hospital leadership in Ho Chi Minh City and Ho Chi Minh City University of Medicine and Pharmacy (42/GT-DHYD-D).

## 3. Results

### 3.1. Translation and Cross-Cultural Adaptation of the GMAS

After translation and cross-cultural adaptation, there is a high-level equivalence between the pre-final and English versions. The average score was 0.99 points for the semantic equivalence criterion. The idiomatic, experiential, and conceptual equivalence achieved the absolute average score (1.00 points) (Appendix A). The pre-final version comprises 11 questions in which the first, fourth, and sixth questions have two options. These questions would choose the most suitable option after conducting pilot testing.

Regarding the pilot testing, there were 42 participants (21 males, 21 females; mean ± SD age 58.52 ± 6.70 years). The score for clarity and comprehension of first question (1a and 1b) was 9.95 ± 0.22, that for question four (4a and 4b) was 10.00 ± 0.00, and that for the whole GMAS was 9.98 ± 0.06 (if we choose option 6a) or 9.91 ± 0.09 (if we choose option 6b) (Appendix B). The Vietnamese version of the GMAS was established after pilot testing and adjusting based on patient opinions (Appendix C).

### 3.2. Psychometric Validation of the GMAS

#### 3.2.1. Patients’ Demographic Characteristics

**Figure 1:**
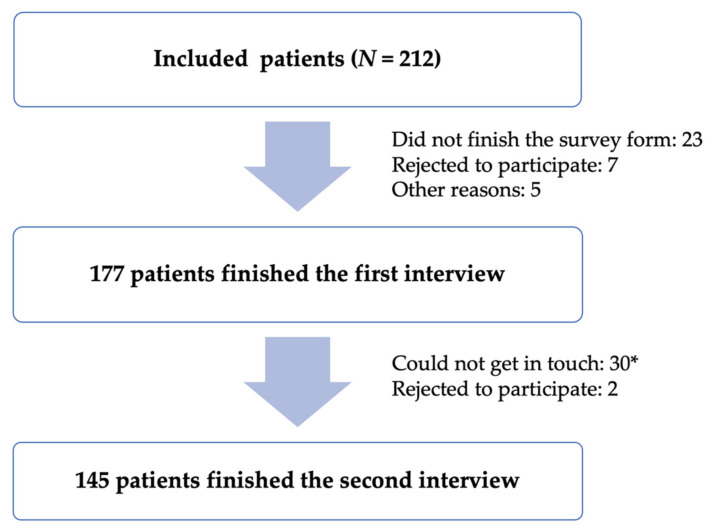
Sample selection and tracking process. (*) The patient will be considered not contacted if the researcher calls seven times without answering (the first day: three times every 30–60 min and four consecutive days thereafter: once a day) or if the patient reads the wrong phone number.

One hundred and seventy-seven patients agreed to participate and finished the first interview. Of these 177 patients, 145 participated in the second interview (Figure 1).

One hundred and seventy-seven patients participated in the study: 67.8% were females, the average age was 59.40 ± 8.67, 93.8% were aged 45 years and over, and 68.9% were not working. Most of the patients participating in the study had an education from high school or higher (90.4%). The patients with a monthly income of 5–10 million were the highest percentage (70.6%). Most (82.5%) of the patients had at least one comorbid condition, 43% had type 2 diabetes for less than five years, and 57% had type 2 diabetes for five years or more (Table 1).

#### 3.2.2. Internal Consistency

Cronbach’s alpha coefficients for the whole scale were 0.817. This figure for each domain was 0.731 (non-adherence due to patient behavior), 0.686 (non-adherence due to other disease and pill burden), and 0.700 (non-adherence due to financial constraints). The corrected item-total correlation of each question was greater than 0.3 (Table 2).

#### 3.2.3. Test–Retest Reliability

Spearman’s correlation coefficients of total medication adherence scores between the results of the first and second interviews two weeks apart were 0.879 with *p* < 0.001. For each question, this figure was higher than 0.600 with *p* < 0.001 (Table 3).

#### 3.2.4. Content Validity

The Vietnamese version was assessed as having high equivalence to the English version by the expert committee in terms of four criteria (semantic, idiomatic, experiential, and conceptual equivalence).

#### 3.2.5. Construct Validity

There was a significant difference (*p* < 0.05) in the proportion of patients’ medication adherence with occupational groups and monthly income levels in the study. In addition, there was a significant difference between the mean score of the BMQ Specific-Necessity and medication adherence (*p* < 0.05) (Table 4). We also analyzed the difference between medication adherence for five levels of medication adherence and patients’ characteristics; similar results were confirmed (Appendix D).

## 4. Discussion

The English version of the GMAS was translated and cross-culturally adapted to create the Vietnamese version. In this process, the sixth question of the scale was specifically adjusted to be more appropriate for type 2 diabetes patients. The English version had no instructions for patients to answer questions. After discussing with the author, he permitted the research team to write an instruction answer for the scale. The pilot testing was conducted to evaluate the level of clarity and comprehensibility of the instruction. After that, it would become the official instruction of the complete Vietnamese version used to assess reliability and validity.

Cronbach’s alpha coefficients for the overall scale were 0.817. This figure changes between 0.7 and 0.9, which shows that there was good internal consistency [18]. The GMAS score with Cronbach’s alpha varied between studies; indeed, in some studies related to the development and validation of the GMAS, Cronbach’s alpha coefficients were 0.840 and 0.797 [10,19]. In addition, the studies featuring the translation and validation of the English version in Pakistan or validation of the GMAS in Saudi Arabia, this figure was 0.819 and 0.74, respectively [11,20]. Two reasons could explain the difference between the figures above. Firstly, the Vietnamese version was adjusted to suit the socio-economic condition, culture, and the target population in Vietnam (the sixth question was modified to be more appropriate for patients with diabetes). Secondly, Cronbach’s alpha coefficients are representative of the survey population [21]. Although Cronbach’s alpha coefficient was affected by the above factors, all these values were >0.7. This showed that the GMAS scale had good consistency with the Vietnamese version and other versions. It was accepted that the GMAS scale was universally applied in many countries.

For the test–retest, the first and second interviews were separated two weeks apart. Spearman’s correlation coefficient was 0.879 (*p* < 0.001), which demonstrates that the Vietnamese version gained stable reliability. Regarding the validation of the GMAS studies (two interviews four weeks apart), this figure was 0.996 (*p* < 0.01) or 0.861 (*p* < 0.01). For the validation of the GMAS in osteoarthritis patients (two interviews three weeks apart), this figure was 0.875 (*p* < 0.001) [12,13,22,23]. In these studies, Spearman’s correlation coefficients indicate that the GMAS has good stability; even though the time between the two interviews was different, the GMAS was still at a good correlation. In addition, the results present the score that each question between two interviews has from moderate to strong correlation (no value less than 0.600). In some cases, patients had different answers, but the total adherence score was still the same. It led to Spearman’s correlation coefficients for the overall scale still being highly correlated. Consider the first interview: for the fifth question, patients chose “always” i.e., “0 points,” and for the sixth question, patients chose “never,” i.e., “3 points”. In the second interview, for the fifth question, patients chose “never,” i.e., “3 points”, and for the sixth question, patients chose “always,” i.e., “0 points”. The total score of both times was 27, although the answers to each question were different in the two interviews. Thus, Spearman’s correlation coefficients for the overall scale and each question indicate that the GMAS has highly stable reliability.

The GMAS shows a considerable difference in adherence and non-adherence between different groups of patients, which indicates the GMAS attained construct validity. There was a significant difference in the proportion of medication adherence between occupational groups (*p* = 0.012). Regarding the officer group, the adherence rate was very high (91.7%). However, the adherence rate of the trader group was low (43.8%). We recorded that most traders were busy all day during the study process, so it was difficult to remember to take medicine. Occasionally, they did not forget to take medication, but there was an interruption at work. The officer group could acquire a lot of knowledge about the disease, so they were more interested in taking medicine and medication adherence. The not-working group mainly was the elderly (retired or homemakers). Consequently, they had more time to attend to their health and tended to have more medication adherence.

There was a difference in medication adherence level between monthly income levels (*p* = 0.015). Patients with income from 3 to 5 million had a high rate of non-adherence, reaching 69.25%. According to patients, some subjects were less likely to pay for the medicines or could only afford one of the prescribed drugs. In contrast, others admitted to forgetting to take medicine because of their intense focus on work. This shows that monthly income was related to medication adherence. Our research result was similar to Akiyo Nonogaki’s and Zahraa Mallah’s studies (2019), the proportion of non-adherence in patients with the lowest income level reached 84.4% (*p* < 0.001) and 64.2% (*p* = 0.004), respectively [24,25]. In addition, the average score of BMQ Specific-Necessity was a discrepancy between medication adherence and non-adherence (*p* = 0.001). Specifically, the average score of the adherence non-adherence group was 20.77 ± 2.70 and 18.51 ± 4.24, respectively. This presents the group of adherence patients who knew that medicine was more important and necessary than the non-adherence group.

The Vietnamese version was translated and cross-culturally adapted from the English version of the GMAS. These two versions had a high equivalence level for four criteria that the expert committee evaluated in stage IV of translation and cross-cultural adaptation. Undeniably, the English version achieved content validity in the author’s study [12]. Therefore, the Vietnamese version reached content validity.

### Strengths and Limitations

The translation and cross-cultural adaptation of the GMAS were conducted in Vietnam for the first time. The scale has achieved the criteria of reliability and validity and could be used as a valuable tool in medication adherence studies. It also helps to investigate the causes of poor adherence (such as treatment costs). Furthermore, it will be easier for users to contact the author and publish research results in the future. However, the study has some limitations. Factor analysis was not tested. Further studies using the Vietnamese version of the GMAS could consider factor analysis. Atta Abbas Naqvi developed the GMAS in 2018. Therefore, there have not been many studies related to the translation and validation of this scale.

## 5. Conclusions

During the research process, the study team obtained the Vietnamese version with 11 questions. The scale achieved reliability and validity and had a high equivalence to the English version, which the expert committee assessed. All questions were clear, easy to understand, and suitable for patients in Vietnam. The Vietnamese version was used to measure medication adherence, determine which factors related to medication adherence, and the causes of poor medication adherence. After that, it is recommended for appropriate interventions (patient counseling programs, health care) as well as evaluation of the effectiveness of the intervention on medication adherence.

## Figures and Tables

**Table 1:** Patients’ demographic characteristics.

Characteristics	Number (*n* = 177)	Percentage
Age		
Mean ± SD	59.40 ± 8.67	
<45	11	6.2
45–64	120	67.8
≥65	46	26.0
Gender		
Female	120	67.8
Male	57	32.2
Occupation		
Not working	122	68.9
Officer	24	13.6
Trader	16	9.0
Unskilled labor	15	8.5
Education level		
Elementary school	17	9.6
High school	103	58.2
College/university/higher	57	32.2
Monthly income (million VND)		
3–5	13	7.3
5–10	125	70.6
>10	39	22.1
Duration of diabetes (years)		
<5	76	43.0
5–10	45	25.4
>10	56	31.6
Chronic comorbidity		
Yes	146	82.5
No	31	17.5
Type of comorbidity		
Hypertension	99	67.8
Dyslipidemia	82	56.2
Ischemic heart disease	26	17.8
Treatment with insulin		
Yes	45	25.4
No	132	74.6
BMQ–V (mean ± SD)		
Specific necessity	19.99 ± 4.48	
Specific concerns	12.97 ± 2.11	

BMQ–V, Beliefs about Medicines Questionnaire—Vietnamese version; SD, standard deviation; VND, Vietnamese dong.

**Table 2:** Internal consistency.

Domains/Subscale	Item	Corrected Item-Total Correlation	Cronbach’s Alpha If Item Deleted	Cronbach’s Alpha for Each Domain	Cronbach’s Alpha for the Total Scale
Non-adherence due to patient behavior (intentional and unintentional)	1	0.579	0.793	0.731	0.817
	2	0.385	0.811		
	3	0.481	0.803		
	4	0.352	0.813		
	5	0.765	0.771		
Non-adherence due to additional disease and pill burden and	6	0.570	0.794	0.686	
	7	0.516	0.800		
	8	0.507	0.802		
	9	0.430	0.808		
Non-adherence due to financial constraints	10	0.362	0.813	0.700	
	11	0.351	0.814		

**Table 3:** Spearman’s correlation coefficients.

Item	Spearman’s Correlation Coefficient	*p*-Value
1	0.887	<0.001
2	0.883	<0.001
3	0.756	<0.001
4	0.807	<0.001
5	0.905	<0.001
6	0.898	<0.001
7	0.804	<0.001
8	0.826	<0.001
9	0.711	<0.001
10	0.696	<0.001
11	0.714	<0.001
For all item	0.879	<0.001

**Table 4:** The relationship between medication adherence and patients’ characteristics (two levels of adherence).

Characteristics	Adherence		Non-Adherence		*p*-Value ^a^
	Number	%	Number	%	
Age group (year)					0.207
<45	9	81.8	2	18.2	
45–64	81	67.5	39	32.5	
≥65	26	56.5	20	43.5	
Gender					0.425
Female	81	67.5	39	32.5	
Male	35	61.4	22	38.6	
Occupation					0.012
Not-working	78	63.9	44	36.1	
Officer	22	91.7	2	8.3	
Trader	7	43.8	9	56.2	
Unskilled labor	9	60.0	6	40.0	
Education level					0.756
Elementary school	10	58.8	7	41.2	
High school	67	65.0	36	35.0	
College/university/higher	39	68.4	18	31.6	
Monthly income (million VND)					0.015
3–5	4	30.8	9	69.2	
5–10	83	66.4	42	33.6	
>10	29	74.4	10	25.6	
Duration of diabetes (year)					0.549
<5	50	65.8	26	34.2	
5–10	32	71.1	13	28.9	
>10	34	65.5	22	34.5	
Chronic comorbidity					0.895
Yes	96	65.8	50	34.2	
No	20	64.5	11	35.5	
Treatment with insulin					0.205
Yes	26	57.8	19	42.2	
No	90	68.2	42	31.8	
BMQ-V (mean ± SD)					
Specific-Necessity	20.77 ± 2.70 18.51 ± 4.24				0.001
Specific-Concerns	12.91 ± 2.05 13.07 ± 2.24				0.612

BMQ–V, Beliefs about Medicines Questionnaire–Vietnamese version; SD, standard deviation; VND, Vietnamese dong. ^a^ Chi-square (χ^2^) was used to analyze the impact between patients’ characteristics and medication adherence; Mann–Whitney U-test was used to analyze the impact of belief about medicines on medication adherence.

## Data Availability

Data sharing not applicable.

## Appendix A

**Table A1:** Evaluation Score of the Expert Committee for the Second GMAS (after Adjustment).

Question	Semantic Equivalence	Idiomatic Equivalence	Experiential Equivalence	Conceptual Equivalence
1	10/10	10/10	10/10	10/10
2	10/10	10/10	10/10	10/10
3	10/10	10/10	10/10	10/10
4	10/10	10/10	10/10	10/10
5	10/10	10/10	10/10	10/10
6	9/10	10/10	10/10	10/10
7	10/10	10/10	10/10	10/10
8	10/10	10/10	10/10	10/10
9	10/10	10/10	10/10	10/10
10	10/10	10/10	10/10	10/10
11	10/10	10/10	10/10	10/10
Average score	109/110 (0.99)	110/110 (1.00)	110/110 (1.00)	110/110 (1.00)

## Appendix B

**Table A2:** The Score for Clarity and Comprehension of the GMAS of Patients (Stage V).

Question	Score (Mean ± SD)
I (instruction)	9.98 ± 0.15
1a	9.95 ± 0.22
1b	9.95 ± 0.22
2	10.00 ± 0.00
3	10.00 ± 0.00
4a	10.00 ± 0.00
4b	10.00 ± 0.00
5	10.00 ± 0.00
6a	10.00 ± 0.00
6b	8.93 ± 0.75
7	10.00 ± 0.00
8	9.88 ± 0.33
9	9.95 ± 0.22
10	9.98 ± 0.15
11	9.98 ± 0.15
Overall score (6a)	9.98 ± 0.06
Overall score (6b)	9.91 ± 0.09

## Appendix C

**Table A3:** The Vietnamese Version of the General Medication Adherence Scale.

Dưới đây là những câu hỏi liên quan đến việc dùng thuốc điều trị đái tháo đường của ông (bà). Ở mỗi câu hỏi, ông (bà) vui lòng cho biết “mức độ” ứng với tình trạng dùng thuốc thực sự của ông (bà).			
**Câu hỏi**	**Nội dung**		
**1**	Ông (bà) có gặp khó khăn trong việc nhớ dùng thuốc không?		
**2**	Ông (bà) có quên dùng thuốc do lịch trình bận rộn như du lịch, hội họp, đám tiệc, đám cưới, đi nhà thờ/chùa… không?		
**3**	Khi cảm thấy khỏe, ông (bà) có ngưng dùng thuốc không?		
**4**	Ông (bà) có ngưng dùng thuốc khi gặp các tác dụng không mong muốn như khó chịu ở dạ dày… không?		
**5**	Ông (bà) có ngưng dùng thuốc mà không báo cho bác sĩ biết không?		
**6**	Ông (bà) có ngưng dùng thuốc (điều trị đái tháo đường) do phải dùng thêm các thuốc cho bệnh khác không?		
**7**	Ông (bà) có thấy bất tiện để nhớ dùng thuốc vì chế độ thuốc phức tạp không?		
**8**	Trong tháng qua, có khi nào ông (bà) quên dùng thuốc vì bệnh nặng hơn và cần dùng thêm thuốc mới không?		
**9**	Ông (bà) có tự ý thay đổi chế độ thuốc như liều, số lần dùng thuốc trong ngày không?		
**10**	Ông (bà) có ngưng dùng thuốc vì (các) thuốc này không đáng với số tiền bỏ ra không?		
**11**	Ông (bà) có gặp khó khăn để mua (các) thuốc vì chúng đắt tiền không?		
**Mỗi câu hỏi có 4 mức lựa chọn: luôn luôn (0 điểm), thường xuyên (1 điểm), thỉnh thoảng/đôi khi (2 điểm), không bao giờ (3 điểm).**			
**Tổng điểm tuân thủ tích lũy (cách tính điểm cũ):**			
**Tuân thủ cao**	30–33 điểm	Tuân thủ tốt	27–29 điểm
**Tuân thủ 1 phần**	17–26 điểm	Tuân thủ thấp	11–16 điểm
**Tuân thủ kém**	0–10 điểm		
**Tổng điểm tuân thủ tích lũy theo (cách tính mới):**			
**Tuân thủ**	≥27 điểm		
**Không tuân thủ**	<27 điểm		

## Appendix D

**Table A4:** The Relationship between Medication Adherence and Patients’ Characteristics (Five Levels of Adherence).

Characteristics		*p*-Value ^a^
**Age group (year)**	<45	0.599
	45–64	
	≥65	
**Gender**	Female	0.510
	Male	
**Occupation**	Not working	0.042
	Officer	
	Trader	
	Unskilled labor	
**Education level**	Elementary school	0.540
	High school	
	College/university/higher	
**Monthly income** **(million VND)**	3–5	0.001
	5–10	
	>10	
**Duration of diabetes (year)**	<5	0.335
	5–10	
	>10	
**Chronic comorbidity**	Yes	0.059
	No	
**Treatment with insulin**	Yes	0.519
	No	
**BMQ–V (mean ± SD)**	Specific-Necessity	<0.001
	Specific-Concerns	0.915

BMQ–V = Beliefs about Medicines Questionnaire—Vietnamese version; SD = standard deviation; VND = Vietnamese Dong. ^a^ Chi-square (χ^2^) was used to analyze the impact between patients’ characteristics and medication adherence; Mann–Whitney U-test was used to analyze the impact of belief about medicines on medication adherence.
